# NUTRI-REAPED study: nutritional assessment of French critically ill children and nutrition practice survey in French-speaking pediatric intensive care units

**DOI:** 10.1186/s13613-019-0493-z

**Published:** 2019-01-22

**Authors:** Aurélien Jacquot, Frédéric Victor Valla, Thibault Mura, Lyvonne Nicole Tume, Héléna Bertet, Carole Ford-Chessel, Christophe Milesi, Gilles Cambonie, Arnaud De Luca, Bénédicte Gaillard-Le Roux

**Affiliations:** 1 Pediatric Intensive Care, Arnaud de Villeneuve University Hospital, 371 avenue du doyen G Giraud, 34295 Montpellier Cedex 5, France; 2 Pediatric Intensive Care, CarMEN INSERM, UMR 1060 Equipe INFOLIP, Hospices civils de Lyon, Hôpital Femme Mère Enfant, 59 bd Pinel, 69500 Lyon-Bron, France; 3 Clinical Research and Epidemiology Unit, CIC 1411, University Hospital, Montpellier, France; 4 Faculty of Health and Applied Sciences, University of the West of England, Bristol, BS16 1DD UK; 5 PICU Bristol Children’s Hospital, Upper Maudlin Street, Bristol, UK; 6 Nutrition Unit, INSERM UMR1069, University Hospital of Tours, Tours, France; 7 Pediatric Intensive Care, Hôpital Femme Mère Enfant, CHU de Nantes, 38 boulevard Jean Monnet, 44093 Nantes, France

**Keywords:** Pediatric Intensive care, Malnutrition, Nutrition practice

## Abstract

**Background:**

Impaired nutritional status is adversely associated with suboptimal outcomes in critically ill children. Undernutrition at pediatric intensive care unit (PICU) admission ranges from 15 to 65%. A lack of knowledge of the nutritional status of children in French PICUs prevents us from specifically targeting education. This study aims to describe the nutritional status of children in French PICUs and to assess nutritional practices and physicians’ knowledge of nutrition, in order to focus NutriSIP (the French-speaking PICU nutrition group) future education programs. A prospective observational multicenter point prevalence study was conducted in French PICUs, recruiting all children admitted over three different weeks. Anthropometric measurements were taken (weight, height/length, mid-upper arm, and head circumferences), in order to calculate nutritional indices. Nutritional status was defined according to WHO Body Mass Index *z*-score and dynamic assessment based on growth faltering detection. Concurrently, PICU physicians and PICU nurses from seven French-speaking countries completed a survey to ascertain knowledge about local nutritional care practices and overall nutrition knowledge. PICU physicians’ responses were compared to PICU nurses’ responses (previously published).

**Results:**

Four hundred and thirty-two children were included in the observational study from 27 French PICUs. Undernutrition was diagnosed in 18.5% of them, young age and underlying chronic condition being the two independent risk factors. Faltering growth was diagnosed in 4.8% and overweight in 7.4%. Subjective nutritional assessment was not accurate. Thirty-eight French-speaking PICUs completed the survey. These showed nutritional practices frequently did not comply with international guidelines, especially regarding nutritional goals, and the reasons for withholding enteral nutrition. Comparison between physicians’ and nurses’ responses to the survey showed large discrepancies.

**Conclusion:**

Undernutrition is frequent at admission in French PICUs. Nutritional status should be assessed using a holistic approach, because of the potential impact on outcome. French-speaking PICU healthcare professionals need further nutrition education, in order to improve nutritional practices to comply with international recommendations. This study will serve as a baseline to focus NutriSIP teaching programs in the future.

**Electronic supplementary material:**

The online version of this article (10.1186/s13613-019-0493-z) contains supplementary material, which is available to authorized users.

## Background

Nutritional status at pediatric intensive care unit (PICU) admission is known to affect outcome in critically ill children. Impaired nutritional status is adversely associated with suboptimal outcomes (increased mortality, PICU length of stay, invasive ventilation duration, and rates of acquired infection) [1–3]. Recent American guidelines [4] strongly recommend systematic nutritional status assessment within the first 48 h of PICU admission. This is recommended using a holistic approach [5], combining both static and dynamic measurements, and describing malnutrition in terms of its etiology, severity, mechanisms, chronicity, and impact on outcomes.

Critically ill children often have underlying chronic health conditions, which may in themselves impact on their nutritional status. Moreover, providing nutritional requirements can be challenging in this setting, resulting in nutrient deficits over the duration of PICU stay [1], which may induce in hospital malnutrition, and worsen outcomes. Identifying these children at risk is essential, in order to provide individualized nutrition support.

Large international PICU studies describing nutritional status at admission have been conducted [1, 6], but no published data exist in French-speaking PICUs. Therefore, to investigate this in a large number of French-speaking PICUs, of different types (medical, surgical or mixed, with various levels of training and concern about nutrition care) is important to gain a more accurate overview of baseline practices and to understand physicians and nurses’ knowledge in this area.

NutriSIP (the French-speaking PICU nutrition workgroup) is composed of dieticians, nurses, and physicians from some French-speaking countries (France, Belgium, and Switzerland) involved in PICU nutrition research and education. NutriSIP aims to improve nutritional practices among critically ill children. This study (the “Nutri-ReaPed study”) designed by NutriSIP aimed to describe the nutritional status of children admitted to French PICUs, by recruiting the majority of French PICUs through its network. In parallel, a survey whose aim was to describe PICU practices and knowledge around nutrition was sent to nurses [7] and physicians in the wider French-speaking PICU network. This was to describe compliance with current recommendations and guidelines and to compare knowledge between professional groups. The results of this study will serve as a base for NutriSIP to target its future educational interventions.

## Methods

The Nutri-Reaped study involved three different, but related studies: (1) A PICU nurse survey about their local nutritional practices and knowledge, which was previously published [7]. (2) A PICU physician survey about their local nutritional practices and knowledge. (3) An observational point prevalence study assessing the nutritional status of critically ill children in France. This paper presents the results of the latter two studies. Both surveys were disseminated in seven French-speaking countries, while the observational study was conducted only in France, because of ethical permissions.

### The French-speaking PICU nutrition practice survey

In 2014, healthcare professionals working in 31 French PICUs, and also in another 11 PICUs within French-speaking countries (Belgium, Switzerland, Algeria, Lebanon, and Quebec, Canada) were asked to participate in the nutrition practice survey.

A 69-question survey was sent to a physician representative of each recruited PICU (NutriSIP members were not allowed to answer the survey to prevent selection bias). This survey (see Additional file 1) was the same as the nursing survey [7], except for seven additional questions regarding energy target prescription, which were added to the physician survey only, as nurses in French-speaking countries are not involved in prescribing nutrition goals (The electronic survey had been sent to the lead nurses of each PICUs who were asked to select one nurse confident enough with the local practices to answer the survey; thus, only one physician and one nurse per center answered the survey). The physician survey was tested for face validity on four physicians and then modified slightly to improve clarity prior to survey dissemination. The questions were selected because they corresponded to the main nutritional targets of NutriSIP. These are: (1) to improve nutritional status assessment, (2) to aware of the specific nutritional needs of critically ill children (energy, protein goals) and (3) to optimize feed delivery to meet these nutritional goals. More details regarding the construction and dissemination of this survey are described in the nurse Nutri-Reaped publication [7]. This survey aimed to describe physicians’ practices and knowledge in comparison with published guidelines, but also to be able to compare physicians’ and nursing teams’ responses to assess compliance between physicians and nurses.

### Observational study of nutritional status in French critically ill children

We conducted a prospective observational multicenter point prevalence study in France in 2014 (nutritional status assessment), following ethical approval (CPP Sud-Méditerranée IV). This study was registered on ClinicalTrial.gov (NCT02293434). Thirty-one French PICUs affiliated with the French-speaking PICU scientific society (GFRUP) participated in this study. Participating French units, who admitted children aged 1 month to 18 years, consecutively recruited children over three different, one-week time periods in 2014 (February, June, October), to avoid any seasonal recruitment bias. These units were a mixture of standalone PICUs or mixed (pediatric and adult or neonatology) intensive care units. Children whose parents did not understand French or refused to participate were not included. Anthropometric measurements were taken on each child, within the first 48 h of admission in the PICU, according to WHO guidelines [35]. These were: weight (kg) measured with an accurate scale, according to local practices (if a child could not be weighed for medical reasons, the most recent accurate weight was collected from patient history or questioning parents); height (cm) or length (cm) was measured from skull to heel or estimated from ulna length measurement (for children above 1 m), as described by Gauld et al. [8]. Head and mid-upper arm circumferences (cm) were also measured. A written protocol for conducting these measurements was provided to ensure consistency of measurements. Local investigators (physician or dietician) were also asked to subjectively describe the child as “undernourished,” “well nourished” or “overweight” for age, prior to anthropometric measurements. A dynamic nutritional assessment was also requested, and local investigators were asked to record weight loss in the 3 months prior to PICU admission, and faltering growth, defined as a weight-for-age growth chart presenting with a deceleration of > -− 1 *z*-score in the previous 3 months. Previous anthropometric measurements were obtained from medical records or from personal medical records.

Anonymized anthropometric data further allowed for centralized calculation of nutritional indices (e.g., body mass index BMI) and cohort nutritional status description. BMI-for-age *z*-score was used as a continuous variable to describe nutritional status, using both French and World Health Organization (WHO) references to allow for other cohort comparisons [9]. Undernutrition was defined as a BMI-for-age *z*-score < -2 SD, as per WHO standards, and overweight as a BMI-for-age *z*-score > 2 SD. Nutritional status was further described using other nutritional indices: weight-for-age *z*-score, weight-for-height *z*-score, Waterlow index, height/length-for-age *z*-score, mid-upper arm circumference *z*-score, mid-upper arm circumference/head circumference ratio when age ranges were appropriate [10]. In case of history of prematurity, *z*-score for age were based on the corrected age.

The calculation of nutritional indices was performed using the survey function of WHO software “ANTHRO” and “ANTHROplus,” available online [36] and EPINUT software (www.epinut.fr) an online nutritional assessment tool certified by the French-speaking Society of Clinical Nutrition and Metabolism, for WHO and French references, respectively. These calculations were based on age and gender and anthropometric data collected from the study population, and expressed as *z*-scores, according to recent recommendations [10, 35, 36].

### Statistical analysis

Physicians’ responses and patients’ characteristics are described using median (*Q*1–*Q*3) for quantitative variables and with frequencies and percentages for qualitative variables. Agreement between survey responses from nurses and physicians was analyzed using the Cohen’s Kappa coefficient and its 95% confidence interval. Cohen’s kappa measures the agreement between two raters beyond chance agreement. Agreement was classified according to Landis and Koch. Kappa could be classified as poor (< 0.0), slight (0.00–0.20), fair (0.21–0.40), moderate (0.41–0.60), substantial (0.61–0.80), or almost perfect (0.80–1.00). To examine differences between nurses’ and physicians’ responses, a Chi-square Mac Nemar was used. A Chi-square Mac Nemar with a *p* value < 0.05 implies that a specific response was significantly more frequently obtained in one of the studied healthcare professional subgroups.

Patients’ characteristics were compared according to nutritional status (WHO standards for undernutrition) using Wilcoxon or Kruskal–Wallis tests for quantitative variables, and using Chi-squared test for qualitative variables. We used logistic regression model to calculate the adjusted odds ratios and their 95% confidence intervals. Only variable with a *p* < 0.2 in univariate analysis was entered in the model. The two-sided significance level set at 0.05. SAS^®^ version 9.4 (SAS Institute, Cary, NC) was used.

## Results

### French-speaking PICU nutrition practice survey

Thirty-eight PICUs, out of 43 (88%), completed the physician survey (Algeria 1/1, Belgium 4/4, Quebec, Canada 3/3, France 27/31, Lebanon 1/1, Luxemburg 0/1, Switzerland 2/2). Of these, 18 (48.6%) were mixed neonatology/pediatric ICUs, and 81.7% admitted both surgical and non-surgical children. Fifteen PICUs were small admitting < 400 children a year, 16 were medium-sized admitting 400–800 and seven were large admitting > 800 children annually.

**Fig. 1:**
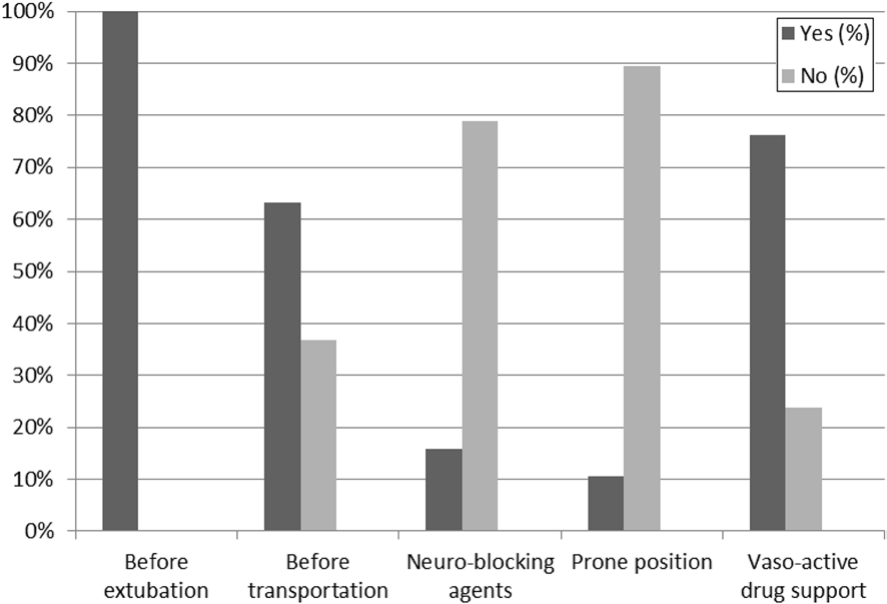
Reasons for withholding enteral nutrition (*n* = 38)

The main survey results are summarized in Table 1 (detailed responses are shown in Additional file 2). Physicians stated that enteral nutrition was initiated within the first 48 h of PICU admission in 90% PICUs. Reasons for withholding enteral feeding are shown in Fig. 1. Gastric residual volume measurement (to guide enteral feeding) was routine practice in 47.4% of units, enteral feeding was administered continuously in 52.6%, prokinetics were frequently used in 57.9%, and transpyloric feeding was never used in 47.4% of units. Supplemental parenteral nutrition (PN) was started on day 1 in 40% of units, and within the first 96 h in almost (96%), if nutritional goals set by the physician could not be met by enteral route. Standardized PN bags with lipids were regularly used in 76.3% of PICUs. Tight glycemic control (defined as glycemia < 1.8 g/L) was followed in 35% of PICUs. Finally, 23.7% of physicians felt confident with the risks, diagnosis, and management of refeeding syndrome. The average agreement on the survey between physicians and nurses [7] within each center was 66% (± 13% with a fair mean kappa = 0.26) (Table 2).

**Table 1:** Physicians’ answers to key nutrition survey questions (*n* = 38)

Questions	*N* (%)
Do you have inside your PICU a physician dedicated to nutrition support?	
Yes	16 (42)
Do you have a dedicated dietician involved in your PICU?	
Yes	22 (58)
Do you have local written nutrition guidelines?	
Yes	20 (53)
How would you assess physicians’ knowledge about nutrition support? *N* = 37	
Poor	19 (50)
Satisfactory	18 (47)
Do you use indirect calorimetry routinely? *N* = 37	
Yes	6 (16)
How do you set energy goals in critically ill children?	
Schofield equations	4 (11)
French National recommended dietary allowance	27 (71)
Others equations	3 (8)
I don’t know	4 (10)
How frequently are children weighed at PICU admission? *N* = 37	
All the time	27 (73)
Sometimes	10 (27)
How frequently is length measured in children (under 1 m)?	
All the time	8 (21)
Sometimes	25 (66)
Never	5 (13)
How frequently is Length/Height measured in children (above 1 m)?	
All the time	4 (11)
Sometimes	21 (55)
Never	13 (34)
When is enteral nutrition usually started? (h)	
< 24	21 (55)
24–48	14 (37)
After 48	3 (8)
When is parenteral nutrition started if enteral/oral nutrition does not fulfill nutrition goals?	
Day 1 (early)	16 (42)
Day 2–4 (early)	18 (47)
Day 5–8 (late)	2 (6)
Day 8 (late)	2 (5)

**Table 2:** Comparison of nurses’ and physicians’ responses

	Cohen’s Kappa^a^ coefficient (IC95%)	Agreement rate %	*p* value for balance of discordant responses^b^	Detailed discrepancy
Is nutrition support considered a priority?	0.29 [− 0.06; 0.64]	80.6	0.53	
Is there a nutrition support team in the unit?	0.47 [0.18; 0.76]*	75,0	0.32	
Is a dietician involved in nutrition care?	0.28 [− 0.03; 0.60]	66.7	0.25	
Are there written local nutrition guidelines?	0.26 [− 0.04; 0.57]	63.9	0.17	
How often are children weighed?	0.12 [− 0.20; 0.44]	*77.4*	*0.03*	Nurses answer “all the time” more frequently
How often is length/height measured?	0.38 [0.13; 0.64]*	65.7	0.20	
How is nutritional status assessed?	0.30 [0.05; 0.55]*	64.5	0.09	
Are nutritional indices (such as BMI) calculated?	0.17 [− 0.05; 0.39]	*55.6*	*0.05*	Physicians answer « no » more often
How are nutritional goals set?	0.03 [− 0.04; 0.10]	*25.7*	*< 0.01*	Nurses answer “I don’t know” more often Physicians answer “recommended dietary allowance” more often
Knowledge about enteral solutions	0.06 [− 0.17; 0.30]	45.7	0.07	Nursing teams consider their knowledge insufficient more often
Use of fibers in enteral nutrition	0.22 [− 0.04; 0.48]	60.0	0.34	
Delay to start enteral nutrition	0.06 [− 0.13; 0.25]	40.0	0.39	
Route and modalities of enteral nutrition	0.36 [0.10; 0.61]*	57.1	0.33	
Patients’ positioning while on enteral nutrition	0.14 [− 0.13; 0.41]	*54.3*	*0.01*	Nurses answer “all the time” more often
Gastric residual volume measurements	0.54 [0.31; 0.77]*	66.7	0.37	
Post-pyloric feeding	0.49 [0.20; 0.78]*	74.3	0.74	
Enteral nutrition withholding prior to extubation	0.00 [− 0.0004; 0.0004]	97.2	0.32	
Duration of enteral nutrition withholding prior to extubation	0.66 [0.39; 0.93]*	85.7	0.65	
Enteral nutrition withholding prior to transport	− 0.01 [− 0.31; 0.29]	55.6	0.13	
Enteral nutrition withholding while neuro-blocking agent use	0.38 [0.11; 0.65]*	*65.7*	*0.04*	Nurses answer « yes » more often
Enteral nutrition withholding while prone positioning	0.34 [0.03; 0.65]*	79.4	0.28	
Enteral nutrition withholding while hemodynamic instability	0.26 [0.05; 0.46]*	*51.4*	*0.04*	Physicians answer “withholding if hemodynamic instability” more often
Use of industrial 3-chamber parenteral nutrition bags	0.12 [− 0.26; 0.50]	79.4	0.26	
Use of standardized parenteral nutrition bags	0.60 [0.33; 0.87]*	*83.3*	*0.01*	Nurses answer « yes » more often
Use of Y-infusion of different parenteral nutrient solutions	0.48 [0.22; 0.75]*	*73.5*	*0.02*	Nurses answer « yes » more often
Micronutrient supplementation	0.35 [− 0.05; 0.75]	68.7	0.81	
Use of individualized compounded parenteral nutrition bags	0.12 [− 0.10; 0.34]	*50.0*	*0.03*	Nurses answer « yes » more often
Pre-op fasting practices	− 0.08 [− 0.33; 0.18]	36.1	0.19	
Post-op fasting practices	0.19 [− 0.12; 0.50]	58.8	0.11	

Agreement is poor (< 0.0), slight (0.00–0.20), fair (0.21–0.40), moderate (0.41–0.60), substantial (0.61–0.80) or almost perfect (0.80–1.00)

Landis and Koch [11]

Data in italics correspond to discordant responses between physicians and nurses (*p* < 0.05)

^*^Cohen’s kappa is significantly different from 0 when the confidence interval (IC95%) do not contain 0. Agreement between raters is significantly greater than chance agreement

^a^Cohen’s kappa measures the agreement between two raters beyond chance agreement

^b^*p* value for MacNemar test < 0.05: discordant answers between nurses and physicians are not balanced

### The nutritional status of children in French PICUs

During the three one-week periods of recruitment, 432 children (from 27/31 French PICUs) were included. Out of 490 eligible patients, 46 were not included because of early death or transfer, or refused parental consent and 12 were excluded because of missing data compromising BMI calculation. Patient characteristics are detailed in Table 3. Weight was measured at admission in 77% of children, and height/length in 60%; they were estimated in remaining children. Thirty-eight percent of children had received nutritional support (enteral or parenteral) before PICU admission.

**Table 3:** Patients’ characteristics (*n* = 432)

	*N*	Total sample *N* (%) or Median (*Q*1–*Q*3)
Age (years)	432	2.9 (0.5–10.6)
Weight (kg)	429	12.5 (6.8–31)
BMI (*z*-score for age)	429	− 0.59 (− 1.69 to 0.45)
Height/length (cm)	432	90.5 (65–137)
Height/length-for-age (*z*-score)	432	− 0.64 (− 1.85 to 0.52)
PIM2 score	415	2.1 (0.8–8.7)
Males	432	251 (58.1)
Chronic underlying condition	426	240 (56.3)
Chronic enteral or parenteral nutrition	428	381 (88.4)
Provenance	431	
Direct admission from home		87 (20.2)
Other hospital units		225 (52.2)
Other ICU		30 (7)
Pediatric emergency		89 (20.6)
Surgical admission	431	154 (35.7)
Planned admission	431	120 (27.8)
Patients having weight measured at admission	432	333 (77.3)
Patients having height/length measured at admission	432	258 (59.9)

Data are presented as medians (*Q*1–*Q*3) or as *N* (%)

**Fig. 2:**
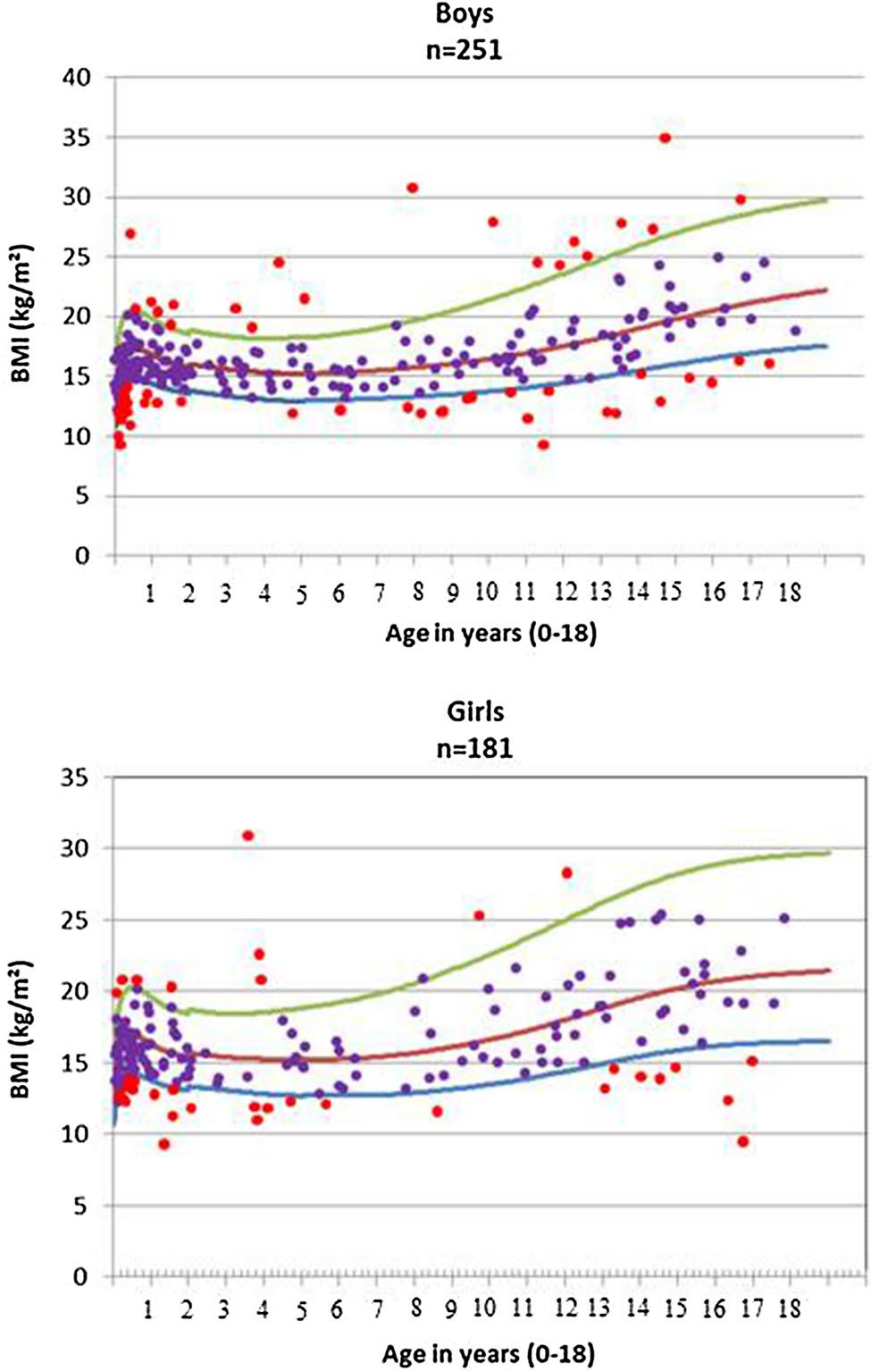
Body mass index (BMI) distribution among girls and boys, according to WHO references (curves represent − 2 SD, mean and + 2SD, respectively). SD: standard deviation for age and gender

According to WHO BMI *z*-score definition, 18.5% of children were diagnosed with undernutrition at PICU admission (Fig. 2). In children under 5 years, undernutrition was diagnosed in 19.6% (CI 95% 14.7–24.5), and those undernourished children were significantly (*p* < 0.001) younger (median age 10 months) than well-nourished children (median age 17 months). In children above 5 years, undernourished children presented more frequently (*p* = 0.004) with a chronic underlying disease (defined as a child presenting with a chronic condition for more than a month). Other nutritional indices are presented in Table 4. Severity of illness (defined by PIM 2 score), surgical admissions or being a planned admission did not significantly affect nutritional status. No significant difference was found between the three time periods of recruitment (*p* = 0.29).

**Table 4:** Nutritional status at PICU admission

Nutritional indices	Age range	Number of children	Values median (IQR) or %
WHO BMI *z*-score	0–18 years	432	− 0.56 (− 1.67 to + 0.46)
French BMI *z*-score	0–18 years	432	− 0.47 (− 1.59 to + 0.71)
WHO BMI *z*-score < − 2SD, undernourished children	0–18 years	432	80 (18.5%)
− 2SD < WHO BMI *z*-score < 2SD, well-nourished children	0–18 years	432	320 (74.1%)
WHO BMI *z*-score > 2SD, overweight children	0–18 years	432	32 (7.4%)
WHO WfH *z*-score	0–5 years	251	− 0.41 (− 1.38 to + 0.71)
WHO WfA *z*-score	0–10 years	321	− 0.76 (− 1.82 to + 0.14)
WHO HfA *z*-score	0–18 years	432	− 0.68 (− 1.85 to + 0.50)
WHO MUAC *z*-score	3 months–5 years	178	− 0.01 (− 1.04 to + 0.84)
French HfA *z*-score	0–18 years	432	+ 0.54 (− 0.83 to + 1.71)
French WfH *z*-score	0–18 years	432	− 0.50 (− 1.50 to + 0.60)
MUAC/HC < 0.3	0–4 years	235	75 (31.9%)
Waterlow indices < 80% (WfH)	0–5 years	251	19 (7.6%)
Waterlow indices < 90% (HfA)	0–18 years	432	64 (14.8%)
Faltering growth	0–18 years	432	19 (4.5%)
5% weight loss within 3 months prior to PICU admission	0–18 years	432	21 (4.8%)

Values are presented as numbers (and percentages) or medians (and 25–75 interquartile)

Waterlow [12]

*WHO* World Health Organisation; French reference; *BMI* Body Mass Index for age; *SD* Standard deviation; *WfH* Weight-for-Height/Length; *WfA* Weight-for-Age; *HfA* Height/Length-for-Age; *MUAC* Mid-Upper Arm Circumference; *HC* Head circumference; *PICU* Pediatric intensive care unit

Table 5 presents the relationship between nutritional status (WHO standards for undernutrition) and patients characteristics. Multivariate analysis showed a higher incidence of undernutrition in children under the age of 1 year (adjusted OR 2.14, CI 95% 1.27–3.61), independently from other confounding factors (underlying chronic condition, type of admission, provenance). Analysis also showed more undernutrition if children were transferred from pediatric emergency units, compared to children with direct admission from home (adjusted OR 3.45, CI 95% 1.42–8.38). In physicians’ subjective assessment of nutritional status, 10% of well-nourished children were considered undernourished, and 67% of children presenting with a BMI-for-age *z*-score < -− 2SD (which defines undernutrition) were considered well nourished.

**Table 5:** Characteristics of patients according to undernutrition status (WHO standards)

	WHO BMI *z*-score < − 2SD Undernutrition *N* = 80^a^	WHO BMI *z*-score > − 2SD No undernutrition *N* = 352^a^	*p* value	AOR^c^ IC95%	*P*^c^ value
Age (years)^b^	1.2 (0.2;10.8)	3.3 (0.6;10.6)	0.04		
Weight (kg)^b^	8.5 (4.1;22.5)	14.0 (7.7;33.0)	< 0.01		
BMI-for-age (*z*-score)	− 2.9 (− 3.6;− 2.4)	− 0.14 (− 1.0;0.75)	–		
Height/length (cm)^b^	83.0 (56;137)	93.7 (67;137)	0.05		
Height/length-for-age (*z*-score)^b^	− 0.56 (− 2.51;0.84)	− 0.69 (1.75;0.45)	0.97		
PIM2 score	2.3 (1.1–15.9)	2.1 (0.8–8.1)	0.28		
Age under 1 years^b^	42 (52.2)	243 (69.0)	< 0.01	2.14 (1.27–3.61)	< 0.01
Males	48 (60.0)	203 (57.6)	0.70		
Chronic underlying condition	51 (64.6)	189 (54.5)	0.10	1.72 (0.96–3.06)	0.06
Chronic enteral or parenteral nutrition	10 (12.5)	37 (10.6)	0.62		
Provenance			0.01		0.02
Direct admission from home	8 (10.0%)	79 (22.5)		1	
Other hospital unit	40 (50.0)	185 (52.7)		1.56 (0.66–3.66)	
Other ICU	9 (11.2)	21 (5.9)		2.62 (0.80–8.55)	
Pediatric emergency	23 (28.7)	66 (18.8)		3.45 (1.42–8.38)	
Surgical admission	125 (35.6)	29 (36.2)	0.91		
Planned admission	28 (35.0)	92 (26.1)	0.11	1.36 (0.72–2.58)	0.34
Patients having weight measured at admission	59 (73.7)	274 (77.8)	0.43		
Patients having height/length measured at admission	49 (61.2)	209 (59.4)	0.75		

Data are presented as medians (*Q*1–*Q*3) or as *N* (%)

*BMI* Body mass index; *WHO* World Health Organization

^a^Sample size without missing data

^b^Because Age, weight and height were highly correlated, we only entered *“Age under 1* *year”* in the multivariate analysis

^c^AOR: Adjusted Odds Ratio (adjusted for Age under 1 year, Chronic underlying condition, Provenance and planned admission)

## Discussion

This is the first prospective study that reports nutritional status of children in pediatric intensive care units in France. The high PICU participation rate allowed for extensive analysis and reduces the risk of recruitment bias. Seasonal recruitment bias was also avoided by collecting data in three time periods. Undernutrition was diagnosed in 18.5% of the children, and those children were significantly younger. Additionally, in our survey, this is the first time that nurses and physicians’ knowledge and practices about nutrition have been compared in French-speaking PICUs. Physicians’ nutritional practices did not comply with international guidance, and their knowledge was inadequate in some areas, while significant differences were found between physicians and nurses’ accounts of local practices.

Undernutrition was frequent and those children were significantly younger. This needs to be taken into consideration by PICU healthcare professionals, as undernutrition is associated with suboptimal outcomes in this setting. This prevalence is higher than those undernutrition rates (10–15%) reported in hospitalized children around Europe (outside PICU) [13–15].

Previous PICU studies showed undernutrition rates at admission ranging between 15 and 20% in Europe, and up to 65% in Brazil [2, 3, 16–18]. These studies, however, were often single center and used different indices to define nutritional status (BMI, Waterlow indices, weight-for-age, height for age, etc.). Two recent international multicenter studies reported undernutrition rates at PICU admission (defined as a BMI *z*-score <  − 2SD) to be 17.1 and 17.9%, respectively [1, 19]. This is close to our results. However, these two studies did not take into account a potential seasonal bias. PICU admission diagnoses are highly seasonal, with respiratory disease prevalent in winter months [20]. This makes comparison with our study challenging. However, Nutri-Reaped study design was underpowered to identify any significant difference between the three time periods of recruitment.

Infants were more likely to be undernourished, as were children transferred from other units, which confirms the vulnerability of children with a medical history and of those with prior chronic conditions leading to PICU admission [21]. The BMI-for-age *z*-score was chosen to define nutritional status, as per WHO recommendations. Many other nutritional indices have been used in the literature, especially weight-for-age *z*-score, which does not require length or height for its calculation. However, such an index does not differentiate undernourished children from “short” stature children and should be interpreted and used with caution. Regarding BMI, like other indices, it will not differentiate lean children from those with undernutrition, and overweight children from those with a muscular body composition.

Faltering growth is a dynamic nutritional assessment recommended by Mehta et al. [5], but is rarely reported in the literature. This was not done in daily practice in most units, and this data cannot be retrospectively extracted from medical files or registries. Its assessment requires plotting values on an appropriate growth chart and the interpretation of this, which is time-consuming. These faltering growth and weight loss rates prior to PICU admission found in our study, are much lower than reported by Valla et al. [22] of 4.8% and 13.7%, respectively. This difference may be attributable to the single center design of the Valla et al. study (with potentially different population recruitment), or the less restrictive definition of growth faltering (weight-for-age curve presenting a deceleration of > − 1 *z*-score in the previous 3 months). However, faltering growth has also been identified in Valla et al. study as a risk factor for increased PICU length of stay and should be actively screened for at PICU admission.

An overweight status was diagnosed in 7.4% of children (BMI *z*-score > + 2 SD), which is lower than the 8.8–10% in the overall French pediatric population [23, 37]. This may be partly due to a higher prevalence of chronic medical conditions in patients admitted to PICU. Being overweight/obese has also been shown to be associated with suboptimal outcomes in various PICU studies while others found no impact. The obesity paradox described in adults is not yet clear so far in critically ill children with conflicting evidence [19, 24].

Finally, the physician’s subjective assessment of children’s nutritional status showed poor reliability compared to anthropometric measurements, especially in the undernourished subgroup, two-thirds of whom were inaccurately categorized as well nourished. This is consistent with previous studies which reported a limited correlation between objective nutritional assessment and subjective assessment, based on trained dieticians and on a detailed tool [25]. Therefore, this subjective assessment of nutritional status cannot be recommended.

The survey revealed the majority of PICU physicians considered nutrition support as a priority (even though this question within a nutrition survey may induce a bias). However, their knowledge about nutritional care was often inadequate, based on their reported practices, compared to current guidelines [4, 26]. Formal nutritional assessment was rarely undertaken and did not comply with the holistic approach recommended by Mehta et al. [5]. Previous work has shown specific PICU training program can be effective [27] to improve anthropometric measurement, and this should be disseminated in other PICUs.

International guidelines recommend that where possible energy requirements should be measured using indirect calorimetry (IC) [4]. However, this device is available in only 15% of PICUs worldwide [28]. Furthermore, a number of PICU clinical conditions prevent the use of IC (27% of patients), such as FiO_2_ > 60%, air leaks, extracorporeal circulation [28]. Predictive equations, specifically Schofield, which are recommended when IC is not possible, were rarely used. In addition, recommended dietary allowances were followed, even though they are known to overestimate energy requirements in this setting, leading to potentially harmful overfeeding (Schofield equations correspond to healthy children’ resting energy expenditure and represent about 65% of recommended dietary allowance). NutriSIP aims to increase physician knowledge and awareness about the optimal method to calculate and prescribe energy goals.

Early enteral nutrition is the preferred administration route, according to current guidelines. However, reasons for withholding enteral nutrition varied between centers, and were not evidence based. [29, 30]. These large variations in responses from PICUs reflect the absence of guidance regarding many practical nutrition delivery issues. One example is the lack of any pediatric studies on prokinetic use in the PICU population. Additionally, the indications for and benefits of post-pyloric feeding on nutrition goal achievement in PICU remain unclear [31, 32]. Local written nutrition guidelines and local nutrition support teams would also help to improve nutritional practices within PICUs [33]. Early parenteral nutrition was standard practice in most PICUs, despite some recent evidence indicating this may be harmful. However, the survey was undertaken prior to the Pepanic trial publication [34]. NutriSIP aims to ensure that nutritional practices in PICU are based on sound evidence or logic, and this is achieved through education programs and future research in this field.

We found some differences and deficits between physicians’ and nurses’ knowledge and practices around nutrition in PICU children. Difference in the education level may explain part of these knowledge differences. However, nurses and physicians also have different roles and responsibilities regarding nutritional care. Physicians are responsible for the nutrition plan (prescription of nutritional support: feeding initiation timing, type of feed, route, and mode of feed delivery, energy, fluid, and protein goals), nurses are responsible for feed administration and feeding tolerance monitoring. Although these roles may sometimes overlap, it is interesting in our study that nurses and physicians sometimes responded differently to the same questions. This may reflect physicians lack of awareness of nursing practices around nutrition. Similarly, nurses frequently lacked awareness of the nutritional strategy planned by physicians. Written guidelines, multiprofessional nutrition rounds, and the continual auditing of practices would help reduce these differences between nurses and physicians. Yet, collaboration between nurses, dietitians, and physicians is essential in the PICU if we are to improve nutritional practices. Training needs to target all three professional groups, but around different areas of knowledge deficiency that is appropriate to their role and responsibilities.

A nutrition support team, consisting of all three professional groups may help achieving this goal. Nurses, especially, need to be engaged in and involved in protocol development, as they are responsible for nutrition delivery. Finally, we suggest review of local professional practices and regular clinical audits of practice after guidelines implementation, in order to ensure compliance with guidelines and direct quality improvement initiatives.

NutriSIP aims to disseminate evidence-based practices in the field of critically ill children nutrition, through research and education projects. NutriSIP will use this survey as a pre-intervention marker of nutrition knowledge and practices in PICUs, which will be reassessed in five years, using the same tool, to evaluate the impact of educational intervention. This intervention consists of an annual one-day free face-to-face teaching program, and open to any healthcare professional involved in PICU nutrition. In addition, various updates are provided in nutrition, at French-speaking pediatric and intensive care congresses. NutriSIP also helps in developing local nutrition guidelines, in order to help physicians setting nutritional goals, and improving nutrition delivery by avoiding unjustified interruptions to enteral nutrition.

This study has some limitations that warrant highlighting. Firstly, weight and height/length could not be measured in all patients; in these, an estimated value was used. This may have impacted on the BMI and other nutritional indices accuracy. Weight accuracy can also be questionable in the PICU setting as it may be influenced by fluid shifts. This may have led to an overestimation of patients’ weight and therefore potentially to an underestimation of undernutrition. Nutritional status definition was based on BMI-for-age *z*-score, as per WHO guidelines, but a holistic approach as defined by Mehta et al. [5] would be required to properly assess nutritional status, including a dynamic assessment, taking into account pathophysiology, etiology, chronicity and the impact of malnutrition. Anthropometric measurements are difficult to perform in the PICU setting: weighing children may be challenging because of PICU equipment (tubes, probes, mechanical ventilation) or considered unsafe; height cannot be measured respecting WHO guidelines in the bedbound child. This may have impacted on measurement accuracy. The diagnosis of faltering growth occurring prior to PICU admission may also be biased by the accuracy and validity of previous anthropometric measurements, performed outside the study. No data regarding outcomes were collected in the study, which did not allow us to assess the nutritional status impact on outcomes. Comparison between countries was not possible as the number of centers was too small in some countries (Switzerland, Lebanon, Algeria, and Canada). Other factors, such as differences in culture, access to healthcare or the geographical location may also have led to the differences in responses. Finally, there is always a risk of self-report bias in surveys, and responding staff was potentially proactive around PICU nutrition issues. Despite these limitations, we have undertaken the largest study of this type in French-speaking PICUs and have achieved a useful baseline upon which to target future interventions. Future research should address determining the optimal height measurement techniques in PICU children.

## Conclusion

Undernutrition in children admitted to French-speaking PICUs is high, especially in infants and in children presenting with chronic underlying medical conditions. PICU professionals’ knowledge is often inadequate and international guidelines are not consistently followed. Assessing nutritional status at PICU admission and following a holistic assessment approach is the basis upon which to implement evidence-based nutrition goal setting to develop individualized nutrition plans. PICU healthcare professionals in French-speaking countries should benefit from increased targeted education and further collaboration, which is a key aim of the NutriSIP Group.

## Additional files

**Additional file 1.** Pediatric Intensive Care Unit (PICU) Physician’s Survey.

**Additional file 2.** Physicians’ answers to the survey.

## Abbreviations

BMI: body mass index
IC: indirect calorimetry
NutriSIP: the French-speaking PICU nutrition workgroup
PICU: pediatric intensive care unit
WHO: World Health Organization
