# Supplementary material for: NUTRI-REAPED study: nutritional assessment of French critically ill children and nutrition practice survey in French-speaking pediatric intensive care units
Source: Ann Intensive Care. 2019 Jan 22;9:15. doi: 10.1186/s13613-019-0493-z (PMC6342745; doi:10.1186/s13613-019-0493-z)
Supplement: Supplementary file 1 — Additional file 1. Pediatric Intensive Care Unit (PICU) Physician’s Survey. [file 13613_2019_493_MOESM1_ESM.doc]

**Pediatric Intensive Care Unit (PICU)**

**Physician’s Survey**

*NUTRI-REAPED*

**Centre:**      

| **INVESTIGATOR**  **COORDONNATor** | **Dr Aurélien JACQUOT**  Pédiatrie Néonatale et Réanimations - Département de Pédiatrie  Pôle Hospitalo-Universitaire "Enfant"  Hôpital Arnaud de Villeneuve - 34295 Montpellier  Tel : 04 67 33 65 73  E-mail : a-jacquot@chu-montpellier.fr |
| --- | --- |
| **PROMOTION** | **Sandrine BARBAS**  CHRU de Montpellier - Direction de la Recherche et de l'Innovation  Hopital La Colombière - Pav.32  39, avenue Charles Flahaut - 34295 Montpellier  Tel : 04 67 33 08 13 |
| **methodologY** | **Dr Thibault MURA**  Centre d'Investigation Clinique, INSERM CIC 1001  Hôpital Saint-Eloi, CHRU Montpellier  80, avenue Augustin Fliche - 34295 Montpellier  Tel: 04 67 33 23 28 |
| **Data MANAGER** | **Héléna BERTET**  Hopital La Colombière - URCE  39, avenue Charles Flahaut - 34295 Montpellier  E-mail : h-bertet@chu-montpellier.fr / Tel : 04 67 33 80 05 |

*How to answer the survey: Select one physician of the PICU team who would be confident enough with the local practices to answer the questionnaire (one questionnaire per PICU).*

*Answers should describe real local current practices (rather than ideal practices) and be related to children’s care (rather than pre-term neonates).*

*It will take about 15 minutes to complete the survey*

1. **You are :**

*If other, specify*:

Your country:

1. **How many years have you been working in** **PICU?**    years
2. **Your PICU is mixed with:**

A NICU (Neonatology ICU):

An Adult ICU:

1. **Number of PICU beds:**
2. **Number of intermediate care beds:**
3. **Number of admission per year :**
4. **Major activity** *(multiple choices)***:**

Surgical patients:

Non-surgical patients:

1. Is your PICU specialized? CiCU, burns, etc.

*(*More than 50% of admissions*)***?**

*If YES,* Type:

*If OTHER, specify*:

**NUTRITIONAL RESOURCES**

1. **Do you have inside your PICU a physician dedicated to nutrition support and trained for this purpose?**
2. **Do you have local written nutrition guidelines available to physicians**

*If YES:*

For enteral nutrition:

For parenteral nutrition:

1. **Do you have written nursing protocols in your PICU?**

*If yes,* For enteral nutrition:

For parenteral nutrition:

1. **Does your PICU have a dedicated nutrition support team?**

*If YES,*  composed of*:*

At least one physician:

At least one nurse:

1. **Do you have a dedicated dietician involved in your PICU?**

*If YES, how often* :

How many dieticians?

If several dieticians, How is their integration in the team:

1. **Does your PICU collaborate regularly with the institutions nutrition support team?**

*If YES, are any PICU members part of this group?*

Are some of the members of this group involved in PICU?

1. **Has your PICU ever participated in a nutrition research program in the past?**

1. **Do you think Nutrition is considered important in your PICU?**
2. **How would you assess physicians’ knowledge about nutrition support in your PICU?**

1. **Do new physicians receive specific nutrition training upon arrival in your PICU?**

**NUTRITIONAL STATUS ASSESSMENT**

1. **Nutritional status assessment in your PICU is based on :** *(only one answer possible)*

A subjective general impression

Growth curves analysis, based on anthropometric measurements

Growth curves analysis, based on anthropometric estimations

I don’t know

1. **Do you routinely use nutritional biomarkers to assess nutritional status?**

*IF YES, which ones (multiple answers)*

Albuminemia:

Bioelectrical impedance (BIA):

Other:

*If YES, specify*:

1. **How frequently are children weighed at admission?**

1. **How frequently is Length measured in children (under 1 meter)?**

1. **How frequently is Length measured in children (above 1 meter)?**

1. **Are malnutrition indices (BMI, WfA, WfH, HfA, etc.) used in routine practice in your PICU?**
2. **In your PICU, assessment of Nutritional status is (only one answer) :**

Systematic at admission (more than 85% of admissions)

Frequent (50 à 84% of admissions)

Not frequent (16 à 49% of admissions)

Rare (less than 15% of admissions)

I don’t know

1. **How often do you monitor nutritional status (weigh, height, etc.) after 5 days of PICU stay :**

**NUTRITION GOALS**

1. **When is parenteral nutrition started if enteral/oral nutrition does not fulfill nutrition goals?**
2. **In children unable to eat orally and with functional digestive tract, what kind of nutritional support do you choose in the first place?**
3. **How do you currently administer enteral nutrition? (only one answer)***:*

Always with an electric pump

Most often with an electric pump, otherwise bolus with gravity

Bolus with gravity most often, sometimes with a pump

Bolus with gravity

I don’t know

1. **Do you measure gastric residual volumes to assess enteral nutrition tolerance?**
2. **Do professionals use the word "gavage" to refer to enteral nutrition? (only one answer)**

Never

Only between healthcare professionals

Always

I don’t know

1. **Do physicians feel confident with children enteral nutrition nomenclature (E.G polymeric, semi-elemental; iso-caloric, 1.0 vs 1.5; age targets, fibers; etc.)?**

1. **Do physicians feel confident with children parenteral nutrition nomenclature? (2 chamber bags, 3 chamber bags, with or without electrolytes, age targets, etc)**

1. **Which positioning do you recommend during enteral feeding infusion?**

*(only one response)*

30-45° or anti trendelenburg

No special patient positioning

I don’t know

1. **How do you set PICU energy goals in critically ill children, when indirect calorimetry is not available?** *(only one answer)*

Schofield equations

Other equations

French National recommended dietary allowance

I don’t know

1. **Do you use indirect calorimetry in your PICU?**

*If YES, which indirect calorimeter*:

1. **In your PICU, in case of conflict between energy and fluid goals (fluid restriction) *(only one response)***

Fluid restriction is more important than energy goals

Energy goals are more important than fluid restriction (diuretics are used if necessary)

Fluid and energy goals are both taken into account (concentrated products, use of diuretics)

I don’t know

1. **In the obese child, would you consider nutrition support less important?**

1. **Is your team familiar with the consequences of over feeding in PICU?**

1. **Do you usually compensate for micronutrient losses and
   amino acids during renal replacement therapy?**

1. **In your PICU, in case of « end of life situations » (treatment withholding or withdrawing project) :**

Artificial nutrition (enteral and/or parenteral) is considered as a treatment and can be withhold/withdrawn

Artificial nutrition is never stopped

1. **If oral nutrition is impossible, when would enteral nutrition be started?**

1. **In the acute phase, how is enteral nutrition preferentially administered?**

1. **Do you use pro-kinetics drugs :**

1. **In children above 3 months, when do you use semi elemental products?:** *(only one answer)*

Only in case of specific indication (pancreatitis, chronic enteropathy ...)

Easily, in case of poor tolerance of polymeric mixtures

I don’t know

1. **Through which route is enteral nutrition most of the time administered?**
2. **Do you use post pyloric tubes :**

1. **Do you use the double gastric tube technique (a suction tube and a feeding tube):**

1. **Do you stop enteral nutrition before extubation :**

*If YES,* When:

1. **Do you stop enteral nutrition before transportation (exam, scan, etc) :**

*If YES, When*:

1. **For children over 12 months, enteral products with fibers are usually prescribed?**

*If YES,* Are fibers discontinued in case of diarrhea:

1. **Do you discontinue or reduce enteral nutrition in case of :***(multiple answers)*

Neuro-blocking agent use:

Morphine use:

Prone positioning:

1. **Is hemodynamic instability a limit for enteral nutrition :** *(only one response)*

Yes, enteral nutrition is stopped in case of use of vasoactive drugs

Enteral nutrition is stopped only if vasoactive drug are not stabilized (increasing doses)

No

I don’t know

1. **You choose to propose a gastrostomy when the duration of
   enteral nutrition exceeds**

*If other, specify*:

1. **For parenteral nutrition, do you use?** *(multiple choice)*

Industrial multi-chamber bags:

Industrial individualized bags:

Pharmacy individualized compounded bags:

Separate bottles (lipid bag, amino acid solution, etc.):

Reconstitution of a single bag by nurses from separate bottles at bedside

1. **Do you frequently use peripheral parenteral nutrition :**
2. **What types of lipid emulsion do you use in parenteral nutrition :** *(multiple answers)*

INTRALIPIDE®:

MEDIALIPIDE®:

SMOFLIPID®:

OMEGAVEN®:

1. **What would contra indicate intravenous lipid infusion? (multiple answers)**

Thrombocytopenia:

Hemophagocytosis:

Uncontrolled sepsis:

Any sepsis:

Other*:*

*If YES,* specify:

1. **Do you sometimes supplement pharmaco-nutrients**

**(e.g. oméga3, anti-oxydants, glutamine)?**

*If YES, which one (multiple answers)*:

Oméga3:

Anti-oxydants:

Glutamine:

Other*:*

*If YES, specify*:

How? (Multiple answers)*)* :

Enterally:

Parenterally:

1. **Do you sometimes add nutrients to parenteral nutrition bags** *:*

Electrolytes and minerals:

*If YES, they are added*:

Micronutrients (vitamins, trace elements):

*If Yes, they are added*:

Other:

*If Yes, specify*:

They are added:

1. **In a stable child receiving parenteral nutrition, how often do you monitor tolerance (plasma samples)?**

*If Other, specify*:

**NUTRITION PRESCRIPTION**

1. **Who is responsible for daily nutrition prescriptions in your PICU?**

Junior physician:

Senior physician:

Dietician:

Nursing staff*:*

Other*:*

*If YES, specify*:

1. **What is your attitude concerning glycemic control?**

1. **If a child encounters hyperglycemia while receiving parenteral nutrition (which currently fulfill energy goals) what do you recommend**

Total energy intake reduction:

Insulin infusion:

**SPECIAL medical conditions**

1. **What is your practice for pre-operative fasting? :** *(only one response)*

Systematically the night before

Systematically at midnight

Systematically 6 hours before surgery

Systematically 4 hours before surgery

Depends on the type of feeds

I don’t know

1. **Does your PICU have a pre-operative « oral clear liquids maintenance » policy?**
2. **Postoperatively, oral or enteral nutrition is started :** *(only one answer)*

Once the bowel opens (gas and / or stool)

Regardless of bowel opening

I don’t know

1. **In case of a chylothorax, you recommend :** *(only one answer)*

No restriction

Classic oral or enteral nutrition

Oral or enteral nutrition with restricted long chain triglycerides

Total parenteral nutrition

I don’t know

1. **Refeeding syndrome :** *(only one answer)*

Physicians are not aware of this syndrome

Is subject to a prevention and management protocol available in the PICU

I don’t know

***Thank you for your participation.***
