# Supplementary material for: NUTRI-REAPED study: nutritional assessment of French critically ill children and nutrition practice survey in French-speaking pediatric intensive care units
Source: Ann Intensive Care. 2019 Jan 22;9:15. doi: 10.1186/s13613-019-0493-z (PMC6342745; doi:10.1186/s13613-019-0493-z)
Supplement: Supplementary file 2 — Additional file 2. Physicians’ answers to the survey. [file 13613_2019_493_MOESM2_ESM.docx]

**Physicians’ answers to the survey**

| **QUESTIONS** | N (%) |
| --- | --- |
| **1. NUTRITIONAL RESSOURCES** | |
| **Do you have inside your PICU a physician dedicated to nutrition support and trained for this purpose?** | 16 (42) |
| **Do you have local written nutrition guidelines in your PICU available to physicians?** | 20 (53) |
| For enteral nutrition | 14 (70) |
| For parenteral nutrition | 19 (95) |
| **Do you have written nursing protocols in your PICU? For enteral nutrition** | 13 (87) |
| **Do you have written nursing protocols in your PICU? For parenteral nutrition** | 10 (67) |
| **Does your PICU have a dedicated nutrition support team?** | 15 (40) |
| Composed of: At least one physician | 10 (67) |
| Composed of: At least one nurse | 12 (80) |
| **Do you have a dedicated dietician involved in your PICU?** | 22 (58) |
| More than once a week | 5 (23) |
| Less than once a week | 8 (36) |
| Every working day | 9 (41) |
| **Does your PICU collaborate regularly with the institution’s nutrition support team?** | 5 (13) |
| **Has your PICU ever participated in a nutrition research program in the past?** | 8 (21) |
| **Do you think Nutrition is considered important in your PICU?** | 31 (82) |
| **How would you assess physicians’ knowledge about nutrition support in your PICU?** |  |
| Good | 3 (8) |
| Insufficient | 34 (90) |
| **Do new physicians receive specific nutrition training upon arrival in your PICU?** | 28 (74) |
| **2. NUTRITIONAL STATUS ASSESSMENT** | |
| **Nutritional status assessment in your PICU is based on:** |  |
| A subjective general impression | 10 (26) |
| Growth curves analysis, based on anthropometric measurements | 22 (58) |
| Growth curves analysis, based on anthropometric estimations | 6 (16) |
| **Do you routinely use nutritional biomarkers to assess nutritional status?** | 13 (34) |
| Albuminemia | 13 (100) |
| Bioelectric impedancemetry (BIA) | 13 (100) |
| **How frequently are children weighed at admission?** |  |
| All the time | 27 (73) |
| Sometimes / hardly ever | 10 (27) |
| **How frequently is Length measured in children (under 1 meter)** |  |
| All the time | 8 (21) |
| Sometimes / hardly ever | 25 (66) |
| **How frequently is Length measured in children (above 1 meter)?** |  |
| All the time | 4 (11) |
| Sometimes / hardly ever | 21 (55) |
| **Are malnutrition indices (BMI, WfA, WfH, HfA, etc) used in routine practice in your PICU?** | 10 (26) |
| **In your PICU, assessment of Nutritional status is** |  |
| Systematic at admission (more than 85% of admissions) | 3 (8) |
| Frequent (50 to 84% of admissions) | 12 (31) |
| Not frequent (16 to 49% of admissions) | 14 (37) |
| Rare (less than 15% of admissions) | 9 (24) |
| **How often do you monitor nutritional status (weigh, height, etc.) after 5 days of PICU stay** |  |
| Once a week or more | 23 (60) |
| Less than once a week | 15 (40) |
| **3. NUTRITION GOALS** | |
| **When is parenteral nutrition started if enteral/oral nutrition does not fulfil nutrition goals?** |  |
| Day 1 | 16 (42) |
| Day 2-4 | 18 (48) |
| Day 5-8 | 2 (5) |
| After day 8 | 2 (5) |
| **In children unable to eat orally and with functional digestive tract, what kind of nutritional support do you choose in the first place?** |  |
| Enteral nutrition | 37 (97) |
| Parenteral nutrition | 0 (0) |
| Glucose infusion | 1 (3) |
| **How do you currently administer enteral nutrition?** |  |
| Always with an electric pump | 29 (76) |
| Most often with an electric pump, otherwise bolus with gravity | 9 (24) |
| bolus with gravity most often, sometimes with a pump | 0 (0) |
| Bolus with gravity | 0 (0) |
| **Do you measure gastric residual volumes to assess enteral nutrition tolerance?** |  |
| All the time | 18 (47) |
| Sometimes | 16 (42) |
| Never | 4 (11) |
| **Do physicians feel confident with children enteral nutrition nomenclature (e.g. polymeric, semi-elemental; isocaloric, 1.0 vs 1.5; age targets, fibers; etc.)?** | 22 (58) |
| **Does the medical team feel confident with children parenteral nutrition nomenclature (2-chamber-bags, 3-chamber-bags, with or without electrolytes, age targets, etc.)?** | 31 (82) |
| **Which position do you recommend during enteral feeding infusion?** |  |
| 30-45° or anti trendelenburg | 17 (45) |
| No special patient position | 21 (55) |
| **How do you set PICU energy goals in critically ill children, when indirect calorimetry is not available?** |  |
| Schofield equations | 4 (11) |
| Other equations | 3 (8) |
| French National recommended dietary allowance | 27 (71) |
| **Do you use indirect calorimetry in your PICU?** | 6 (16) |
| **In your PICU, in case of conflict between energy and fluid goals (fluid restriction)** |  |
| Fluid restriction is more important than energy goals | 5 (13) |
| Energy goals are more important than fluid restriction | 2 (5) |
| Fluid and energy goals are both taken into account | 31 (82) |
| **In the obese child, would you consider nutrition support less important?** | 4 (11) |
| **Is your team familiar with the consequences of over feeding in PICU?** | 13 (34) |
| **Do you usually compensate for micronutrient losses and amino acids during renal replacement therapy?** | 9 (24) |
| **In your PICU, in case of « end of life situations » (treatment withholding or withdrawing project):** | 36 (95) |
| Artificial nutrition is considered as a treatment and can be withhold/withdrawn | 23 (64) |
| Artificial nutrition is never stopped | 15 (68) |
| **If oral nutrition is impossible, when would enteral nutrition be started?** |  |
| Within the first 24h | 21 (55) |
| Within 24-48h | 14 (37) |
| After 48h | 3 (8) |
| **In the acute phase, how is enteral nutrition preferentially administered?** |  |
| Bolus | 3 (8) |
| Continuous feeding | 20 (53) |
| Bolus + continuous feeding | 15 (39) |
| **Do you use pro-kinetics drugs?** | 22 (58) |
| **In children above 3 months, when do you use semi elemental products?** |  |
| Only in case of specific indication (pancreatitis, chronic enteropathy ...) | 14 (37) |
| Easily, in case of poor tolerance of polymeric mixtures | 18 (47) |
| **Through which route is enteral nutrition most of the time administered?** |  |
| Gastric | 36 (95) |
| Post pyloric | 2 (5) |
| **Do you use post pyloric tubes?** | 20 (53) |
| **Do you use the double gastric tube technique (a suction tube and a feeding tube)?** | 9 (24) |
| **Do you stop enteral nutrition prior to extubation?** | 38 (100) |
| Several hours prior to extubation | 26 (68) |
| Upon extubation | 12 (32) |
| **Do you stop enteral nutrition before transportation?** | 24 (63) |
| Several hours prior to transportation | 2 (9) |
| Upon transportation | 20 (87) |
| **For children over 12 months, enteral products with fibers are usually prescribed?** | 18 (47) |
| **Do you discontinue or reduce enteral nutrition in case of?** |  |
| Neuro-blocking agents | 6 (16) |
| Morphine use | 0 (0) |
| Prone positioning | 4 (11) |
| **Is hemodynamic instability a limit for enteral nutrition** |  |
| Yes, enteral nutrition is stopped in case of use of vasoactive drugs | 4 (10) |
| Enteral nutrition is stopped only if vasoactive drugs are not stabilized (increasing doses) | 25 (66) |
| No | 9 (24) |
| **You choose to propose a gastrostomy when the duration of**  **enteral nutrition exceeds** |  |
| 1 month | 9 (24) |
| 2 months | 2 (5) |
| 3 months | 8 (21) |
| Other | 10 (26) |
| **For parenteral nutrition, do you use?** |  |
| Industrial multi-chamber bags | 29 (76) |
| Industrial individualized bags | 11 (29) |
| Pharmacy individualized compounded bags | 23 (62) |
| Separate bottles (lipid bag, amino acid solution, etc.) | 17 (46) |
| Reconstitution of a single bag by nurses from separate bottles at bedside | 12 (32) |
| **Do you frequently use peripheral parenteral nutrition?** | 16 (42) |
| **What type of lipid emulsion do you use in parenteral nutrition** |  |
| INTRALIPIDE® | 13 (36) |
| MEDIALIPIDE® | 17 (46) |
| SMOFLIPID® | 23 (61) |
| OMEGAVEN® | 3 (8) |
| **What would contra indicate intravenous lipid infusion?** |  |
| Thrombocytopenia | 6 (16) |
| Hemophagocytosis | 26 (70) |
| Uncontrolled sepsis | 25 (68) |
| Any sepsis | 2 (6) |
| **Do you sometimes supplement pharmaco-nutrients (oméga3, anti-oxydants, glutamine)?** | 4 (11) |
| Oméga3 | 2 (50) |
| Anti-oxydants | 1 (25) |
| Glutamine | 0 (0) |
| Enterally | 2 (50) |
| Parenterally | 3 (75) |
| **Do you sometimes add nutrients to parenteral nutrition bags** |  |
| Electrolytes and minerals | 22 (58) |
| Micronutrients (vitamins, trace elements) | 23 (62) |
| **In a stable child receiving parenteral nutrition, how often do you monitor tolerance (plasma samples)?** |  |
| Once a week | 23 (60) |
| Twice a week | 12 (32) |
| Every 2 days | 1 (3) |
| Every day | 2 (5) |
| **4. NUTRITION PRESCRIPTION** | |
| **Who is responsible for daily nutrition prescriptions in your PICU** |  |
| Junior physician | 36 (95) |
| Senior physician | 35 (97) |
| Dietician | 9 (25) |
| Nursing staff | 2 (6) |
| **What is your attitude concerning glycemic control?** |  |
| Tight glycemic control | 13 (35) |
| No tight glycemic control | 13 (35) |
| No pre-defined protocol | 11 (30) |
| **If a child encounters hyperglycemia while under parenteral nutrition infusion (which currently fulfill energy goals) what do you recommend** |  |
| A restriction of energy intake | 14 (37) |
| Insulin infusion | 29 (78) |
| **5. SPECIAL MEDICAL CONDITIONS** | |
| **What is your practice for pre-operative fasting** |  |
| Systematically the night before | 0 (0) |
| Systematically at midnight | 1 (2) |
| Systematically 6 hours before surgery | 20 (53) |
| Systematically 4 hours before surgery | 5 (13) |
| Depends on the type of feeds | 12 (32) |
| **Does your PICU have a pre-operative « oral clear fluid maintenance » policy?** |  |
| No | 24 (63) |
| Sometimes | 4 (11) |
| Often | 7 (18) |
| Always | 3 (8) |
| **Postoperatively, oral or enteral nutrition is started** |  |
| Once the bowel opens (gas and / or stool) | 20 (54) |
| Regardless of bowel opening | 17 (46) |
| **In case of a chylothorax, you recommend** |  |
| No restriction | 0 (0) |
| Classic oral or enteral nutrition | 1 (3) |
| Oral or enteral nutrition with restricted long chain triglycerides | 30 (79) |
| Total parenteral nutrition | 7 (18) |
| **Refeeding syndrome** |  |
| Physicians are not aware of this syndrome | 29 (76) |
| Is subject to a prevention and management protocol available in the PICU | 7 (18) |
